# Unenhanced magnetic resonance imaging for the evaluation of sonographically indeterminate ovarian and adnexal masses

**DOI:** 10.1590/0100-3984.2024.0032

**Published:** 2025-02-07

**Authors:** Behnaz Moradi, Maryam Aghasi, Maryam Rahmani, Elham Sharifi, Mahrooz Malek, Fariba Yarandi, Masoumeh Banihashemian, Nadereh Behtash, Hamed Abdolghafoorian

**Affiliations:** 1 Tehran University of Medical Sciences, Tehran, Iran; 2 Yas Hospital Complex, Tehran, Iran; 3 Shariati Hospital, Tehran, Iran; 4 Vali-Asr Hospital, Tehran, Iran; 5 Shahid Beheshti University of Medical Sciences, Tehran, Iran

**Keywords:** Ovarian neoplasms, Adnexal diseases, Magnetic resonance imaging, Contrast media, Gadolinium DTPA, Ultrasonography, Neoplasias ovarianas, Doenças dos anexos, Ressonância magnética, Meios de contraste, Gadolínio DTPA, Ultrassonografia

## Abstract

**Objective:**

To investigate the accuracy of magnetic resonance imaging (MRI) in classifying sonographically indeterminate ovarian and adnexal masses.

**Materials and Methods:**

This was a retrospective cross-sectional study of the unenhanced pelvic MRI scans of 243 patients with a collective total of 336 adnexal and ovarian masses.

**Results:**

Unenhanced MRI showed a sensitivity of 97.7%, a specificity of 86.4%, and an accuracy of 93.8%. The area under the ROC curve was 0.944 (95% CI: 0.913–0.974).

**Conclusion:**

Our results show that an unenhanced MRI protocol can be used to classify adnexal masses, especially in clinical settings in which the intravenous administration of gadolinium-based contrast is not safe and should be avoided.

## Abstract (pt)

**Objetivo:**

Investigar a precisão da ressonância magnética (RM) na classificação de massas ovarianas e anexiais indeterminadas na ultrassonografia.

**Materiais e Métodos:**

Este foi um estudo transversal retrospectivo de exames de RM pélvica sem contraste de 243 pacientes com um total coletivo de 336 massas anexiais e ovarianas.

**Resultados:**

A RM sem contraste mostrou sensibilidade de 97,7%, especificidade de 86,4% e precisão de 93,8%. A área sob a curva ROC foi 0,944 (IC 95%: 0,913–0,974).

**Conclusão:**

Nossos resultados mostram que um protocolo de RM sem contraste pode ser usado para classificar massas anexiais, especialmente em ambientes clínicos nos quais a administração intravenosa de contraste à base de gadolínio não é segura e deve ser evitada.

## INTRODUCTION

Ovarian cancer is the most lethal gynecologic cancer and the fifth leading cause of cancer-related death in women. There were approximately 313,959 new cases and 207,252 related deaths worldwide in 2020^(1^, ^2)^. In a recent report, it was estimated that, in the United States alone, approximately 19,710 new cases of ovarian cancer would be diagnosed and 13,270 women would die from the disease in 2023^(2)^.

Late diagnosis due to nonspecific or nonexistent clinical symptoms in the early stages is one of the causes of the high mortality associated with ovarian cancer. Although ultrasound is the first-line imaging modality to examine ovarian masses, magnetic resonance imaging (MRI) plays an essential role as a next-step examination to characterize adnexal lesions for which the ultrasound findings were inconclusive^(3)^.

The Ovarian-Adnexal Reporting and Data System (ORADS) lexicon for ultrasound, which was published in 2018, has been reported to have excellent diagnostic accuracy, with an area under the curve ranging from 0.91 to 0.98^(4)^. However, approximately 5–20% of adnexal masses remain unclassifiable or indeterminate on ultrasound^(5)^. Because of its high accuracy, MRI is the imaging modality of choice for these indeterminate lesions. The new ORADS MRI risk stratification system, introduced in 2020, is a classification system for adnexal masses that has been used as a comprehensive system in numerous studies, with reported sensitivity and specificity values of 91.1—93.0% and 91.0–94.92%, respectively^(6^, ^7^, ^8)^. In a meta-analysis including 4,520 adnexal masses, the O-RADS MRI score was found to have a sensitivity and specificity over 90% for the characterization of adnexal lesions^(8)^.

The O-RADS MRI stratification system is based on the injection of contrast agent and the acquisition of dynamic contrast-enhanced sequences. However, it may be necessary to avoid the use of contrast in some patients and in certain clinical scenarios. It has been posited that the use of a gadolinium-based contrast agent (GBCA) is associated with complications such as nephrogenic systemic fibrosis and other complications caused by the accumulation of gadolinium in the tissues^(9^, ^10^, ^11)^. However, the bio-distribution of these agents appears to be more complex than previously believed. The clearance rate of GBCAs and their safety depend on renal function and their retention would be higher in the setting of impaired kidney function. It is also noteworthy that GBCAs are divided into two categories (linear and macrocyclic) on the basis of the shape of the organic ligand. Because macrocyclic GBCAs are safer, the use of linear GBCAs, and therefore the risk of complications, has been reduced worldwide.

The American College of Radiology guidelines for GBCA administration advise against administration of group I and group III agents (which are in the linear category) in patients on dialysis or with chronic kidney disease stage 4 or 5, to avoid the development, albeit rare, of nephrogenic systemic fibrosis^(12)^.

Another issue that should be considered is the use of GBCAs in pregnant women. According to a recent review of the literature, the safety of GBCA administration during pregnancy, especially during the first trimester, remains unclear. However, some studies have reported no significant differences in outcomes between infants who were exposed to GBCAs and those who were not. The situation of each pregnant woman should be examined individually to weigh the advantages and disadvantages for the mother and the fetus^(13)^.

In clinical practice, there are some patients who refuse contrast agents and others in whom their use is contraindicated, such as those with advanced-stage chronic kidney disease. In such cases, unenhanced pelvic MRI should be performed. In pregnant women, especially those in their first trimester, the advantages and disadvantages of contrast-enhanced MRI should be carefully assessed. Unenhanced pelvic MRI may be able to provide satisfactory information regarding the classification of adnexal masses. In a study conducted by Sahin et al. and published in 2021^(14)^, the sensitivity, specificity, and accuracy of unenhanced MRI in examining adnexal masses were 84.9%, 95.9%, and 94.2%, respectively^(14)^.

The purpose of the present study was to investigate the accuracy of unenhanced MRI in classifying ovarian and adnexal masses.

## MATERIALS AND METHODS

This was a cross-sectional, retrospective multicenter study of the pelvic MRI scans of all women who had been referred from three hospitals and a few private imaging centers to a tertiary referral center for imaging, because of indeterminate ultrasound findings, between 2016 and 2021. The inclusion criteria were having undergone surgery for the treatment of an adnexal mass, having postoperative histopathological results available, having been followed for at least one year after surgery, and having follow-up data available. Patients in whom the MRI scans were considered inappropriate or inadequate were excluded. The final sample comprised 336 adnexal masses in 243 patients. The study was approved by the local research ethics committee (Reference no. IR.TUMS.IKHC. REC.1399.535). Throughout the study process, the confidentiality and anonymity of the participant data were respected. Because of the retrospective nature of the study, the requirement for written informed consent was waived.

### MRI protocol

The patients had fasted for at least three hours before undergoing MRI. All images were acquired in a 3.0-T scanner (Discovery CT750; GE HealthCare, Chicago, IL, USA), with a phased array surface coil. The following sequences were acquired: axial, sagittal, and coronal T2-weighted fast spin-echo sequences; axial T2-weighted sequences with fat suppression; and T1-weighted sequences with and without fat suppression. Diffusion-weighted imaging (DWI) sequences were also acquired in the axial plane, with b values of 50, 500, and 1,000 s/mm^2^, which are routinely used in clinical practice. No contrast-enhanced images were available.

### Image analysis

**Figure 1:**
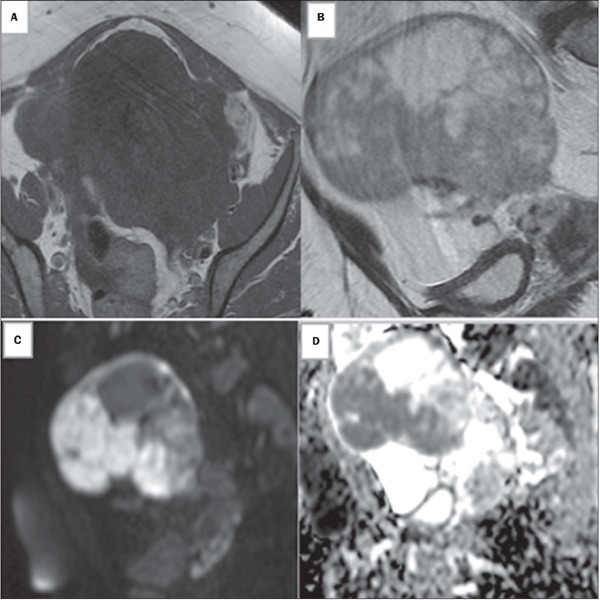
Images of a 50-year-old woman with bilateral adnexal masses categorized as indeterminate on transvaginal ultrasound. Axial T1-weighted and sagittal T2-weighted MRI sequences **(A** and **B,** respectively), showing a left-sided, multiloculated solid-cystic adnexal mass with intermediate signal intensity in its solid portions. DWI sequences showing high signal intensity at b = 1,000 s/mm^2^ **(C)** and low ADC signal intensity in solid tissues **(D).** In the proposed scoring system, based on unenhanced MRI findings, this lesion was given a score of 5 (lymphadenopathy is not shown in the images above). Histopathology confirmed a diagnosis of serous cystadenocarcinoma.

**Figure 2:**
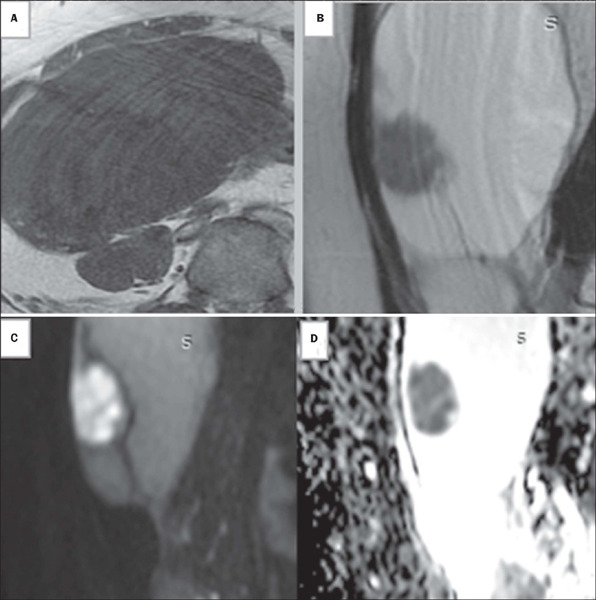
Images of a 50-year-old woman with bilateral adnexal masses categorized as indeterminate on transvaginal ultrasound (the same patient whose left adnexal mass is shown in Figure 1). Axial T1-weighted and sagittal T2-weighted MRI sequences **(A** and **B,** respectively), showing a right-sided, multiloculated solid-cystic adnexal mass with intermediate signal intensity in its solid portions. DWI sequences showing high signal intensity at b = 1,000 s/mm^2^ **(C)** and low ADC signal intensity in solid tissues **(D).** In the proposed scoring system, based on unenhanced MRI findings, this lesion was given an score of 5 (lymphadenopathy is not shown in the images above). Histopathology confirmed a diagnosis of serous cystadenocarcinoma.

Two radiologists, each with at least 10 years of experience in the field of pelvic MRI, studied and interpreted the MRI images on a workstation. Both were working independently and were blinded to the clinical and laboratory findings, as well as to the histopathologic findings and follow-up data. Each radiologist examined approximately half of the cases. Cases of disagreement were resolved by consensus. In cases in which there was more than one adnexal mass, each mass was evaluated separately (Figures 1 and 2). The MRI scans were scored and classified on the basis of the criteria outlined in Table 1, and all assessments of signal intensity were subjective. Lymphadenopathy was defined as a short axis diameter > 8 mm in the iliac lymph nodes and > 10 mm in the para-aortic lymph nodes.

**Table 1:** Diagnostic parameters of the proposed unenhanced MRI scoring system.

Score	Definition	MRI finding
1	No mass	No adnexal mass
2	Benign	Purely cystic masses, fat-containing masses, or endometrioid masses
3	Probably benign/indeterminate	Not classified in other scores
4	Suspicious for malignancy	Solid-appearing tissue with intermediate signal intensity on T2WI, low signal intensity on T1WI, and true diffusion restriction
5	Highly suspicious for malignancy	Peritoneal implants, lymphadenopathy, or ascites after exclusion of benign diagnoses

T2WI, T2-weighted imaging; T1WI, T1-weighted imaging.

True diffusion restriction is defined as high signal intensity on high-b value DWI sequences and low signal intensity on an apparent diffusion coefficient (ADC) map that was assessed subjectively. On DWI, the signal intensity was measured in relation to that of the cerebrospinal fluid on T2-weighted images, whereas the ADC map was evaluated in relation to that of the skeletal muscles. Given that blood-degradation products and fat tissue have low signal intensity on ADC maps due to shortened T1 values, masses such as mature teratoma and endometrioma are common pitfalls in the evaluation of restriction. To avoid such misinterpretation, simultaneously with DWI interpretations, T2-weighted images and T1-weighted images (with and without fat suppression) were evaluated simultaneously with the DWI scans.

### Reference standard

The reference standard in the present study was the histopathological diagnosis. Cases that were not candidates for surgery and in which the histopathological results were not available were subjected to a final evaluation based on clinical monitoring and imaging over the course of at least one year. Tumors regarded as borderline on the basis of the histopathology report were classified as malignant in the statistical analysis.

### Statistical analysis

Data analysis was carried out with the IBM SPSS Statistics software package, version 22.0 (IBM Corp., Armonk, NY, USA). Quantitative variables are reported as mean and standard deviation, whereas qualitative variables are reported as number and percentage. The relationships between the basic patient characteristics and the histopathological results were investigated with the chi-square test. The relationship between the proposed MRI score and the type of adnexal mass was evaluated with the chi-square test or Fisher’s exact test, as appropriate. Diagnostic parameters of scoring systems including sensitivity, specificity, positive predictive value, negative predictive value, and accuracy were calculated, and their validity was evaluated with receiver operating characteristic (ROC) curves.

Values of *p* > 0.05 were considered statistically significant. An MRI score ≥ 4 was adopted as the cut-off value for malignancy. Interobserver agreement was evaluated by using unweighted indices and Fleiss’ kappa.

## RESULTS

A total of 243 patients with 336 adnexal masses were investigated in the present study. The mean age of the patients was 43 years. Approximately 20% of the patients were postmenopausal.

Among the 336 adnexal masses, 196 (58.3%) were treated surgically and follow-up data were available for 140 (41.7%). The masses were categorized as benign, malignant, and borderline in 218 (64.9%), 94 (28.0%), and 24 (7.1%) of the cases, respectively, and 28 (8.3%) were metastatic. None of those 28 cases had an ovarian origin, originating, variously, from gall bladder adenocarcinoma, cervical cancer, breast cancer, rectal cancer, colon cancer, urothelial cancer, and lymphoma. Of the 28 patients with metastatic disease, 18 (64.3%) had another type of cancer concomitantly or had previously received a diagnosis of cancer and had been treated for that cancer. Among the adnexal masses, the mean maximum diameter was 72.6 ± 47.52 mm (range, 7–360 mm) and 62 (18.5%) had an extra-ovarian origin. As shown in Table 2, the most common histopathological type was epithelial.

**Table 2:** Characteristics of the adnexal masses evaluated on unenhanced MRI.

Variable	(N = 336)
Patient age (years), mean ± SD	43 ± 13
Patient menopausal status, n (%)	
Premenopausal	273 (81.3)
Postmenopausal	63 (18.7)
Mass treated surgically, n (%)	196 (58.3)
Follow-up data for the mass, n (%)	140 (41.7)
Mass categorization, n (%)	
Benign	218 (64.9)
Malignant	94 (28.0)
Borderline	24 (7.1)
Largest diameter of the mass (mm), mean ± SD	72.61 ± 47.52
Origin of the mass, n (%)	
Ovarian	274 (81.5)
Extra-ovarian	62 (18.5)
Malignant/borderline mass type, n (%)	
Epithelial	81 (68.6)*
Germ cell	2 (1.7)*
Sex cord	7 (6.0)*
Metastasis	28 (23.7)*

* n = 118 masses (94 categorized as malignant + 24 categorized as borderline).

**Figure 3:**
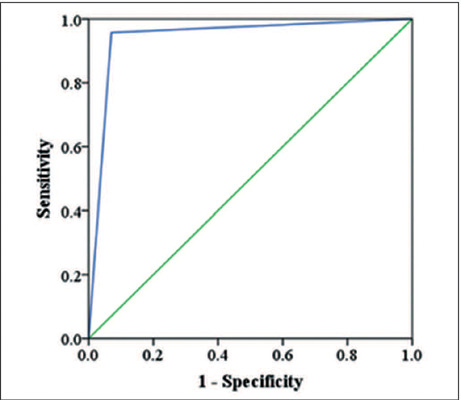
ROC curve of the unenhanced MRI scoring system for adnexal masses, at a cutoff score for malignancy of ≥ 4.

**Figure 4:**
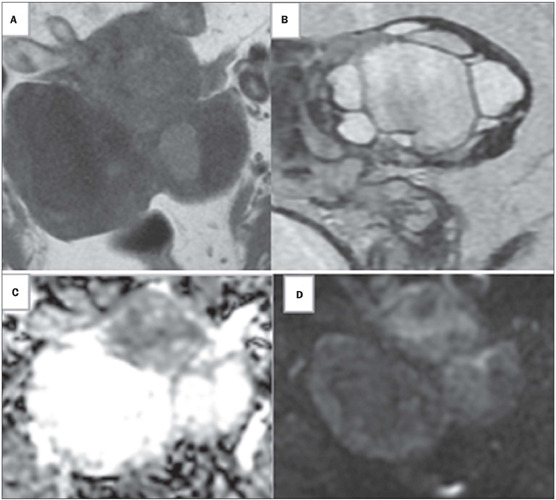
Images of a 59-year-old woman with bilateral adnexal masses categorized as indeterminate on transvaginal ultrasound. Axial Tl-weighted and sagittal T2-weighted MRI sequences **(A** and **B,** respectively), showing a left-sided, multiloculated solid-cystic adnexal mass with intermediate signal intensity in its solid portions. DWI sequences showing high signal intensity at b = 1,000 s/mm^2^ **(C)** and intermediate-to-high ADC signal intensity in solid tissues **(D).** In the proposed scoring system, based on unenhanced MRI, this lesion was given a score of 3. Histopathology confirmed a diagnosis of a borderline serous tumor.

When we considered an MRI cutoff score for malignancy of ≥ 4, unenhanced MRI had sensitivity, specificity, and accuracy values of 97.7%, 86.4%, and 93.8%, respectively (Table 3). As illustrated in Figure 3, the area under the ROC curve was 0.944 (95% CI: 0.913–0.974). Among the 177 masses with an MRI score of 2 (indicative of a benign lesion), two (a serous tumor and a seromucinous tumor) were categorized as borderline on the basis of the surgical and histopathological findings, the MRI score therefore being considered a false-negative result. Among the 52 masses with an MRI score of 3 (probably benign/indeterminate), there were 14 for which the MRI score was considered a false-negative result: two rectal cancer metastases; 10 borderline adnexal masses; one ovarian endometrioid carcinoma; and one differentiated serous carcinoma (Figure 4). Among the 107 masses with an MRI score of 4 or 5 (suspicious or highly suspicious for malignancy), there were (on the basis of the histo-pathology results) five that were categorized as benign—three tubo-ovarian abscesses, one struma-ovarii, and one ovarian fibroma with liquefaction necrosis—representing false-positive MRI results. When we omitted the histopathology-proven borderline cases from the primary data, the sensitivity increased to 95.74%, with a negative predictive value of 98.15 and a negative likelihood ratio of 0.043 (Table 3).The interobserver agreement on the classification of adnexal lesions was almost perfect (kappa = 0.9).

**Table 3:** Diagnostic parameters of scoring systems for adnexal masses.

Parameter	Unenhanced MRI scoring system (this study)		Other scoring systems	
	Including borderline cases	Excluding borderline cases	ADNEX MR (2016-2019)	O-RADS MRI (2020-2022)
Sensitivity (%)	86.4	95.7	93.5-94.9	91.1-93.0
Specificity (%)	97.7	97.7	92.9-96.6	91.0-94.9
PPV	95.3	94.7	94.8	89.1
NPV	93.0	98.2	97.4	95.9
PLR	37.5	41.7	37.5	10.9-18.0
NLR	0.14	0.043	0.05	0.09
Accuracy(%)	93.8	97.1	—	93.7

PPV, positive predictive value; NPV, negative predictive value; PLR, positive likelihood ratio; NLR, negative likelihood ratio.

## DISCUSSION

In our study, the sensitivity, specificity, and accuracy of unenhanced MRI were 86.4%, 97.7%, and 93.8%, respectively. These results are congruent with those of a similar study, published in 2021, in which its sensitivity, specificity, and accuracy were reported to be 84.9%, 95.9%, and 94.2%, respectively^(14)^.

Among the various MRI scoring systems for adnexal masses that have been proposed, two standardized systems have been developed and are used universally: the ADNEX MR score and the O-RADS MRI score^(6^, ^15^, ^16^, ^17)^. Studies published between 2016 and 2019 have shown that the ADNEX MR score has a sensitivity of 93.5—94.9% and a specificity of 92.9—96.6%^(15^, ^16^, ^17)^. The more recently developed O-RADS MRI score has been shown to have a sensitivity and specificity of 91.1-93.0% and 91.0-94.9%, respectively^(6^, ^7^, ^8)^.

Our results are incongruent with the results of the abovementioned studies in terms of the sensitivity of the proposed MRI score, which was found to be less sensitive in our study sample. That is likely due to the fact that some histopathology-proven borderline masses (considered malignant in our statistical analysis) were downgraded to a score < 4 due to a lack of restricted diffusion. When we omitted the histopathology-proven borderline cases from the primary data, the sensitivity increased.

Sixteen histopathology-proven borderline masses were downgraded to an MRI score of 2 or 3. Although more studies are required, it can be concluded that image interpretation is more challenging in borderline tumors than in benign and malignant tumors, as well as that interpretation of a DWI sequence alone without taking the pattern of contrast enhancement into account could lead to underestimation of the risk of malignancy in borderline tumors.

It has been assumed that if the ADNEX MR score is assessed without dynamic contrast-enhancement, the specificity for malignancy would be below 90%^(5)^. Nevertheless, in our study and in a similar study^(14)^, published in 2021, the specificity of unenhanced MRI was found to be 97.7% and 95.9%, respectively.

Our study has some limitations. A one-year follow-up period might have been too short for the efficient monitoring of slow-growing adnexal masses that have not been treated surgically. In addition, the study population might have been too small to allow reliable conclusions to be drawn. Studies with larger samples are needed in order to confirm these results. Furthermore, the signal intensities on DWI sequences were assessed subjectively. However, the images were interpreted by experienced radiologists specializing in gynecological imaging. Therefore, the accuracy of the unenhanced MRI protocol might not be generalizable to populations in which the images are interpreted by general radiologists or radiologists with less experience. Future studies should be designed to address this issue.

## CONCLUSION

Although further studies are needed, the results of the present study show that an unenhanced MRI protocol can be used to classify adnexal masses, especially in certain clinical settings, such as in patients with contraindications to the use of contrast. In addition, the shorter acquisition times in this protocol could save time and reduce the radiology department workload. However, it is extremely important to acquire high-quality DWI images during these examinations.
